# CT pattern analysis of necrotizing and nonnecrotizing lymph nodes in Kikuchi disease

**DOI:** 10.1371/journal.pone.0181169

**Published:** 2017-07-24

**Authors:** Eun Jung Shim, Kyung Mi Lee, Eui Jong Kim, Hyug-Gi Kim, Ji Hye Jang

**Affiliations:** 1 Department of Radiology, Kyung Hee University College of Medicine, Kyung Hee University Hospital, #26 Kyunghee-daero, Dongdaemun-gu, Seoul, Korea; 2 Department of Radiology, Korea Cancer Center Hospital, #75 Nowon-ro, Nowon-gu, Seoul, Korea; Cambridge University, UNITED KINGDOM

## Abstract

**Objective:**

The purpose of this study was to determine whether a CT interpretation with imaging pattern analysis differentiates Kikuchi disease (KD) from the two more frequently encountered differential lymph nodes diagnoses of tuberculous lymphadenopathy (TL) and reactive hyperplasia (RH).

**Materials and methods:**

Between January 2012 and July 2015, 20 patients with KD (6 men, 14 women; mean age, 27.80 years), 36 patients with RH (10 men, 26 women; mean age, 33.08 years) and 34 patients with TL (17 men, 17 women; mean age, 39.82 years) were pathologically diagnosed using US-guided fine needle aspiration biopsy, core needle biopsy, or surgical excisional biopsy. We recorded the total number, location, and size of the affected cervical lymph nodes, and two radiologists reviewed the characteristic imaging findings, including the presence of necrosis, cortical enhancement pattern, perinodal infiltration, conglomeration and nodal calcification, to form a consensus. In addition, we compared two attenuation indices on the nonnecrotic portion of the affected lymph nodes, nodal cortical attenuation (NCA) and the ratio of NCA to the adjacent muscle (NCA/M).

**Results:**

Conglomeration, enhancement pattern and NCA/M values were independent predictive CT features to distinguish KD from RH. Age and enhancement pattern discriminated KD from TL. Only the mean NCA/M value was a statistically significant CT feature (p = .008) in differentiating KD from both RH+TL. The mean NCA/M of KD (1.67 ± 0.20) was significantly higher than that of RH (1.49 ± 0.20) or TL (1.47 ± 0.21).

**Conclusion:**

Our results indicate that in case of nonnecrotic lymphadenopathy, a higher NCA/M index can differentiate KD from RH and TL. In addition, the enhancement pattern according to the degree of necrosis discriminated between KD and TL in the case of necrotic lymphadenopathy.

## Introduction

Kikuchi disease (KD), which is also referred to as histiocytic necrotizing lymphadenitis or subacute necrotizing lymphadenitis, was first described by Kikuchi and Fujimoto et al. in 1972 [1]. The etiology of KD is still unknown, but some reports suggest that viral infections or autoimmune diseases may be the causes [1,2]. KD is a benign form of lymphadenitis and spontaneously recovers within weeks to months, so an exact assessment is important to avoid unnecessary procedures and management [3,4]. However, a definitive diagnosis is often challenging, especially because KD has similar imaging features as other common types of cervical lymphadenopathy like tuberculous lymphadenopathy (TL), reactive hyperplasia (RH) of the lymph node, lymphomas, and metastases.

Many articles have presented an analysis of the imaging findings of KD [1–5]. Typical CT findings of KD are multiple, enlarged lymph nodes with commonly unilateral cervical distributions at levels II–V of the cervical neck, and homogenous enhancement with perinodal infiltration, but these have been nonspecific [4]. Nodal necrosis with heterogeneous attenuation is atypical and with mildly indistinct margins, multiple necrotic foci and a high attenuation of necrotic foci are associated with KD [2]. However, KD mimics other forms of benign lymphadenopathy, such as RH and TL. To the best of our knowledge, there has been no study about CT imaging pattern analysis of necrotizing and nonnecrotic lymph nodes in predicting KD. Therefore, the purpose of our study was to determine CT features that differentiate KD from TL and RH.

## Materials and methods

### Study population

This retrospective study was approved by Kyung Hee University Hospital institutional review board with a waiver for the requirement of informed consent. A total of 159 patients underwent contrast-enhanced neck CT, US-guided fine needle aspiration biopsy (FNAB), or core needle biopsy (CNB) of neck lymph nodes from January 2012 to July 2015. Among them, seven patients with unsatisfactory FNAB reports, 11 patients with metastatic lymph nodes and 49 with no malignant cells according to FNAB results were excluded. Two additional cases were eliminated because of severe motion artifacts on CT scans. Out of the total 90 patients included in the analysis, there were 20 with cytohistologically confirmed KD (6 men, 14 women; mean age, 27.80 years), 36 with RH (10 men, 26 women; mean age, 33.08 years) and 34 with TL (17 men, 17 women; mean age, 39.82 years). The clinical diagnosis of patients with RH was cervical lymphadenitis. The study sample of 90 patients was determined by FNAB (n = 45), CNB (n = 25) or surgical excisional biopsy (n = 20).

### CT (Computed Tomography) imaging protocol

The high-resolution 128-CT (Ingenuity Core 128, Philips Healthcare), 64-CT (Brilliance 64, Philips Healthcare), or 16-CT (LightSpeed 16, GE Healthcare) scanners were employed and 95 ml of nonionic contrast medium (iomeron ^®^ [iomeprol], Bracco, Milan, Italy) power injected intravenously at 2.4 ml/sec into the antecubital vein. The concentration of iodine in this contrast was 300mg per 1ml. Images were acquired 40 seconds after contrast injection and reconstructed to 3mm thickness with no gap. Axial and coronal planes were reconstructed from the skull base to the aortic arch level.

### CT image analysis—Qualitative analysis

**Fig 1:**
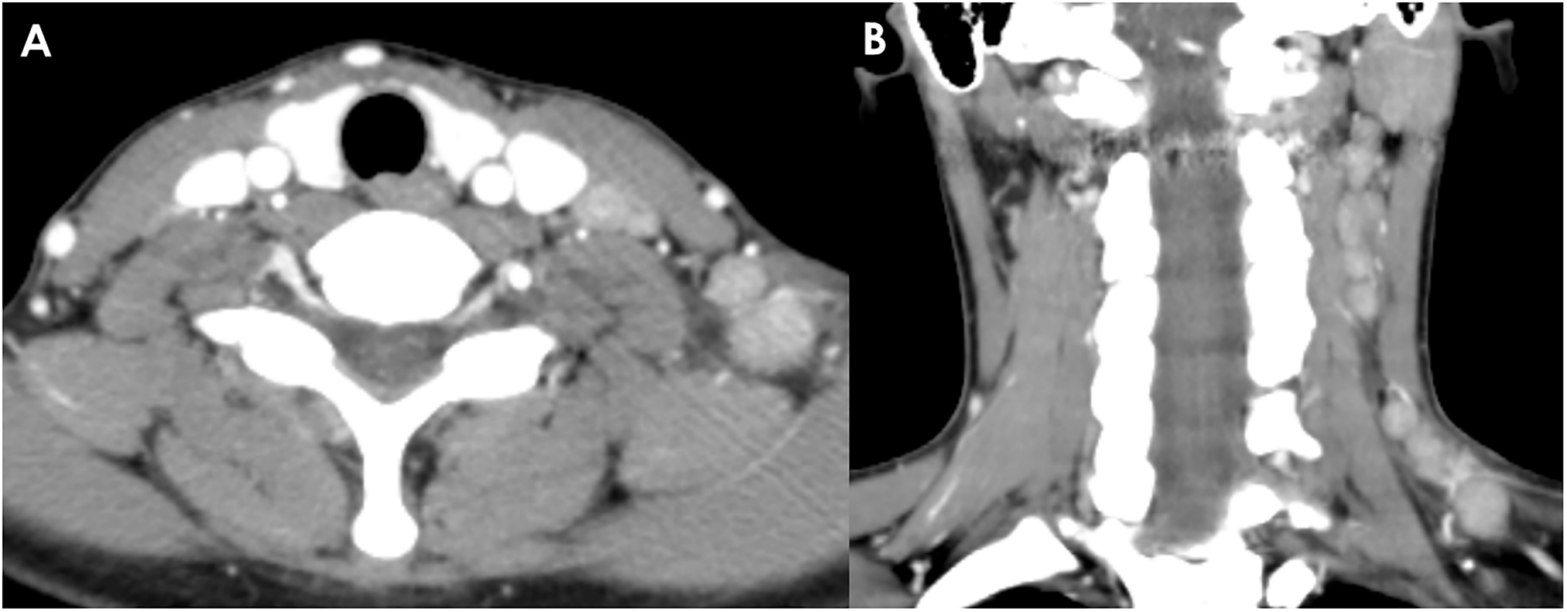
A 27-year-old woman with KD. (A, B) Conglomerated unilateral cervical lymphadenopathies are shown on axial and coronal CT scans, with homogeneous, strong cortical enhancement and mild perinodal infiltration. The mean NCA and NCA/M are 116.0 and 1.71, respectively. The enhancement pattern in this patient is categorized as group 1.

**Fig 2:**
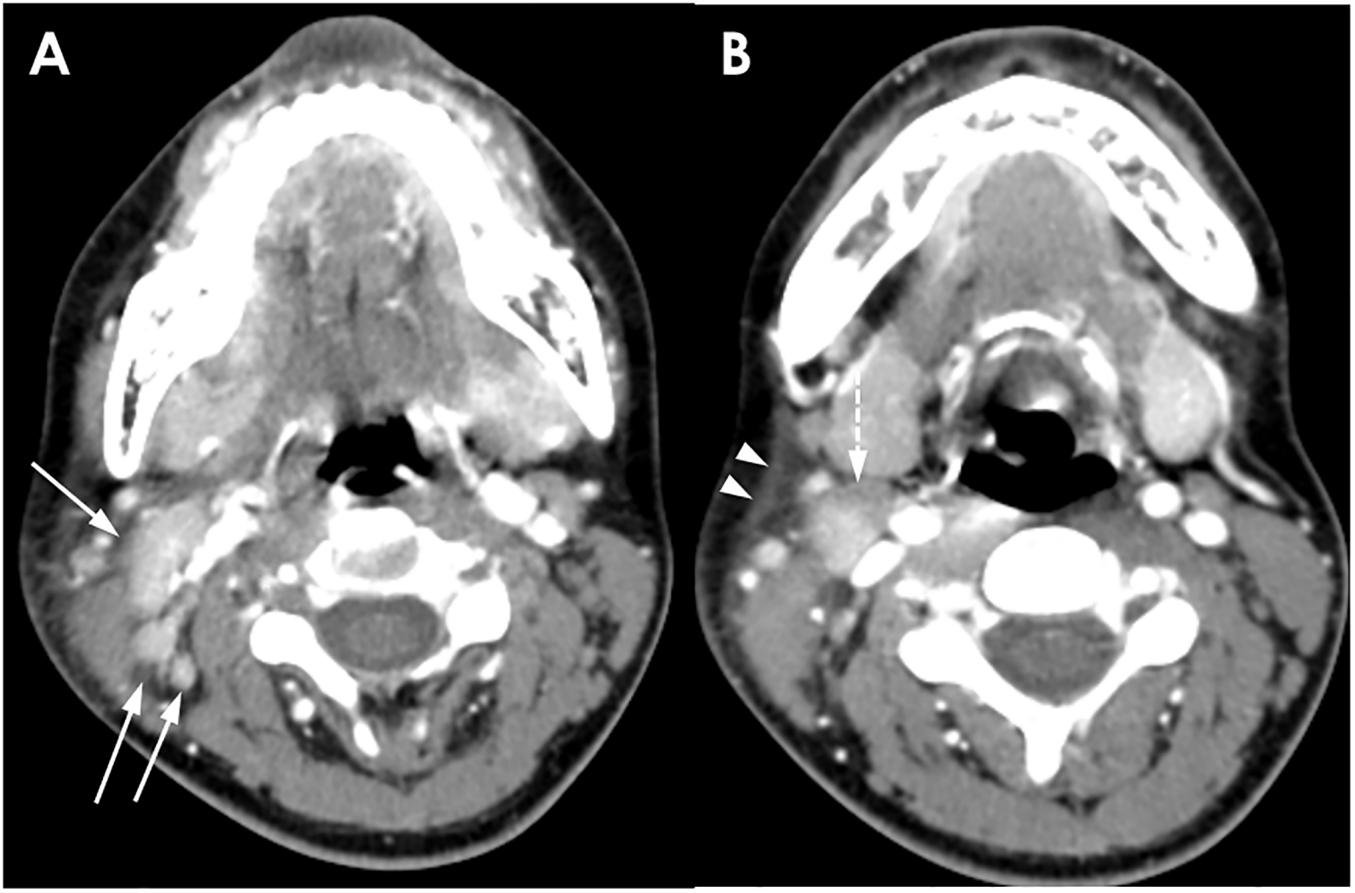
A 25-year-old woman with KD. (A) Conglomerated unilateral cervical lymphadenopathies with prominent cortical enhancement are shown (solid arrows). The mean NCA and NCA/M are 126.0 and 1.94, respectively. (B) Right level II lymph node with a partial necrotic portion (dotted arrow) and perinodal infiltration with associated fascial thickening (arrow heads). This enhancement pattern is categorized as group 2.

**Fig 3:**
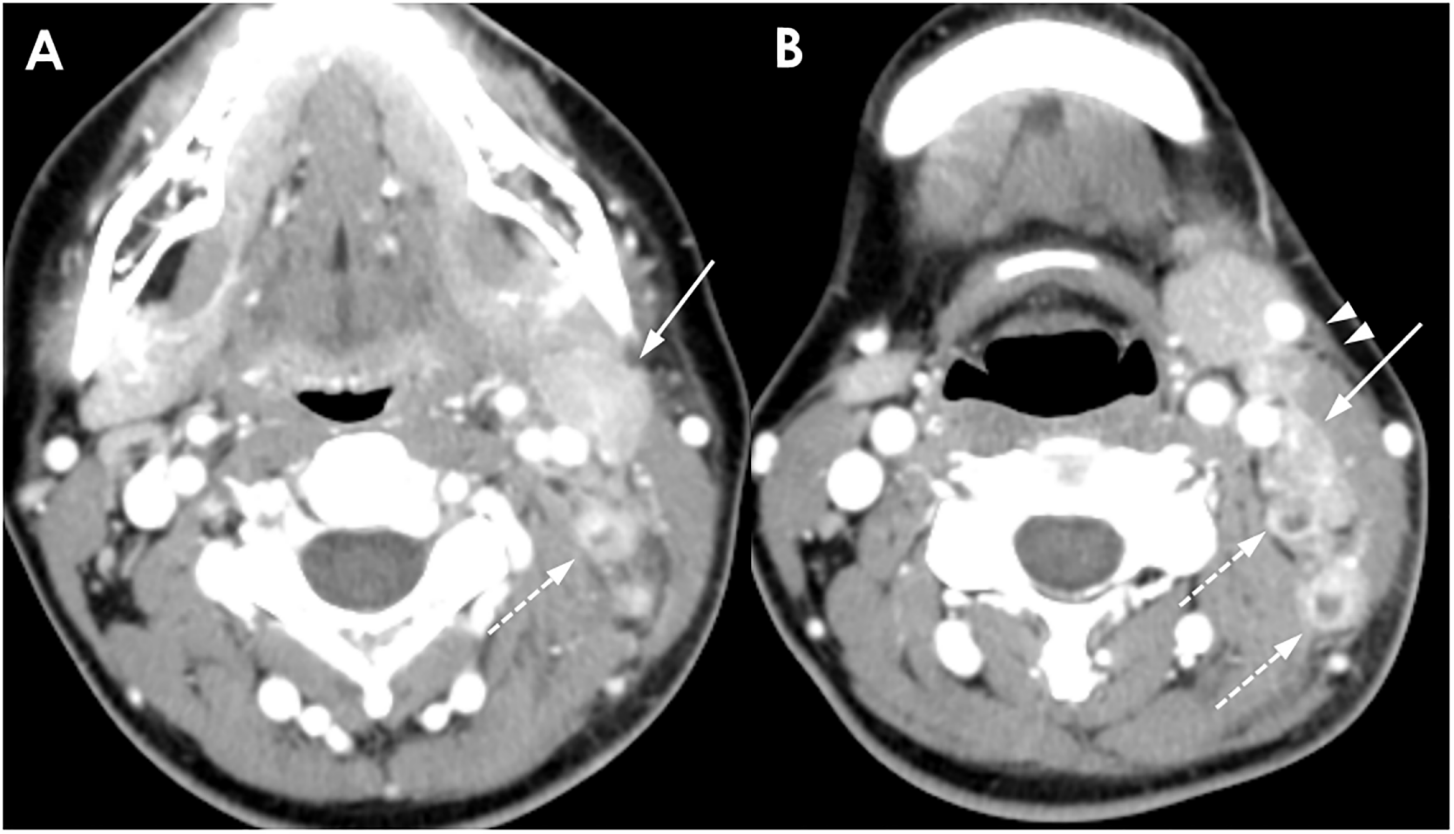
A 35-year-old woman with KD. (A, B) Conglomerated necrotic (dotted arrows) and nonnecrotic (solid arrows) cervical lymph nodes are shown on CT scan. Obvious perinodal infiltration and fascial thickening (arrow heads in B) are noted. The mean NCA and NCA/M are 138.0 and 1.66, respectively. This enhancement pattern is classified as group 3.

**Fig 4:**
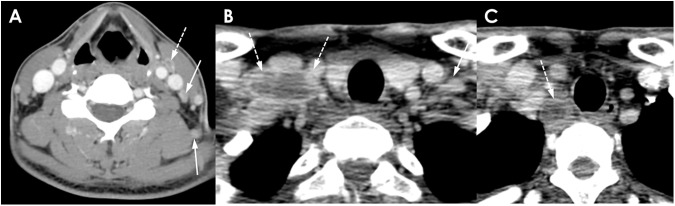
A 36-year-old man with TL. (A-C) Multiple necrotic (dotted arrows) and nonnecrotic (solid arrows) cervical lymphadenopathies with mild perinodal infiltration are shown. The largest necrotic LNs are located in the right supraclavicular area. The enhancement pattern in this patient is classified as group 3. The mean NCA and NCA/M of nonnecrotic LN are 99.7 and 1.33, respectively.

**Fig 5:**
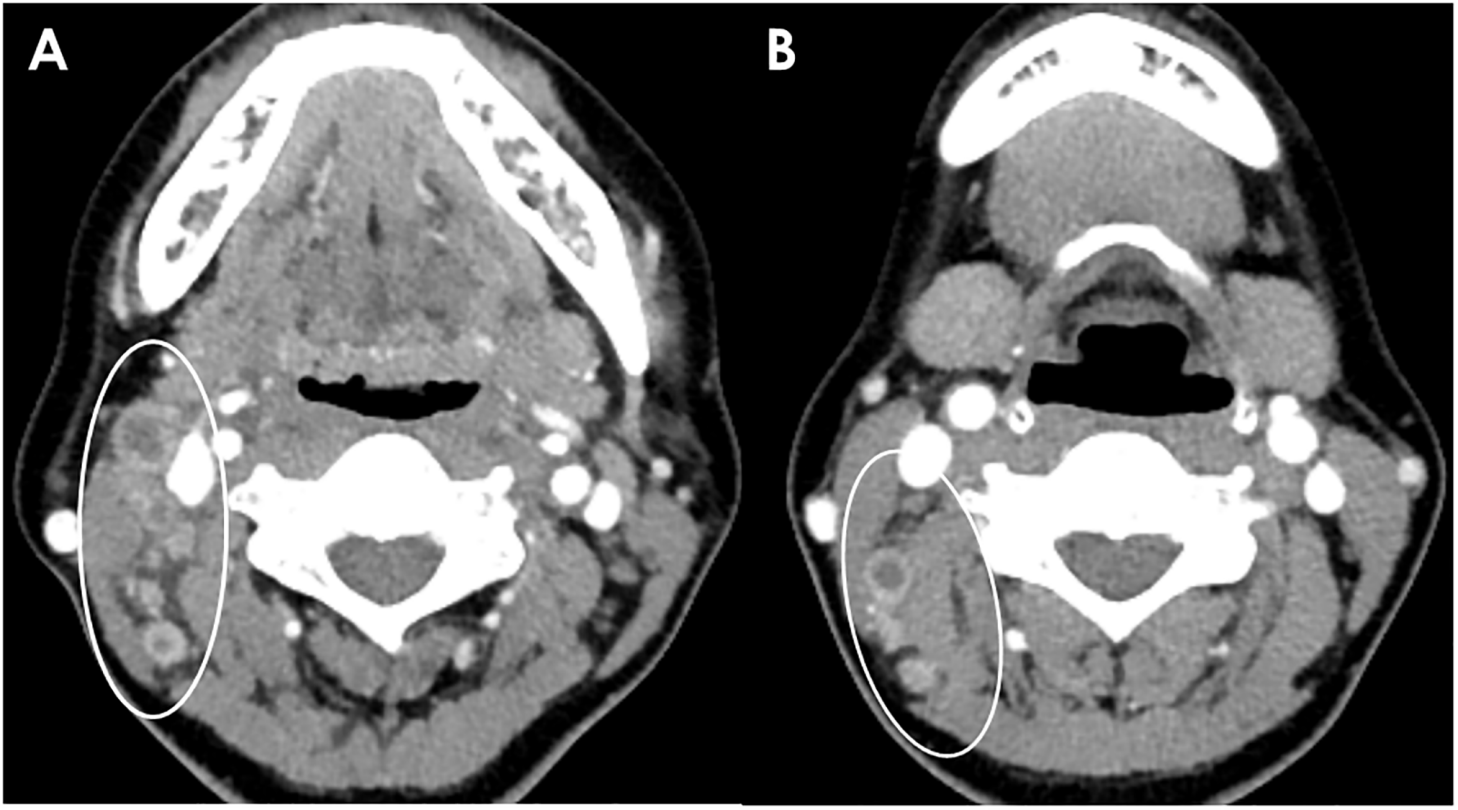
A 46-year-old woman with TL. (A, B) Multiple conglomerated necrotic cervical lymphadenopathies with perinodal infiltration are shown (circle). The enhancement pattern in this patient is categorized into group 4. The mean NCA and NCA/M could not be measured.

Two head and neck radiologists reviewed the CT images to form a consensus at a PACS workstation (IFINITT Healthcare Co., Ltd.) while unaware of the pathologic results. The reviewers recorded the location, size (Long(L)/Short(S) diameter) and shape (L/S ratio) of the affected lymph nodes, as well as characteristics including the presence of necrosis, perinodal infiltration, conglomeration and nodal calcification. Affected lymph nodes were included when one of the following was present: 1) increased size (maximum diameter compared to the normal size of the respective region), 2) abnormal shape (not oblong or lima bean-shaped but spherical), 3) clusters of small lymph nodes, 4) necrosis, 5) perinodal fat infiltration, 6) increased contrast enhancement. Presence of necrosis was considered a focal area of low attenuation on CT scans. The severity of nodal necrosis in a patient was analyzed using a contrast enhancement pattern. The nodal enhancement patterns were categorized into four groups (group 1, nonnecrotic; group 2, <50% of affected nodes; group 3, 50–90%; group 4, >90%) according to the proportion of necrotic nodes among all affected lymph nodes in a patient (Figs 1–5). Perinodal infiltration was considered when lymph nodes showed obliteration of the adjacent fat plane or an increased perinodal attenuation on CT scans. Conglomerate lymph nodes were placed side by side, but could not distinctly be kept apart from each other.

### CT image analysis—Quantitative analysis

The reviewers calculated two attenuation indices of the affected nonnecrotic lymph nodes. The mean nodal cortical attenuation (mNCA) was measured in Hounsfield units (HU) by applying manually-defined circular or oval regions of interest (ROI). The size of the ROI was approximately 5mm^2^. The ROI was measured at the pathologically confirmed lymph node and if it was unknown, at the largest lymph node. We excluded patients whose necrosis was severe in the affected lymph nodes and had limited NCA measurements. In the TL group, 21 patients were excluded and only 13 included for the NCA measurement. We measured at least three regions in the same nodal parenchyma, excluding hilar vessels, and then averaged the values. Furthermore, we calculated the NCA-to-muscle ratio (NCA/M) using adjacent sternocleidomastoid or trapezius muscles. Finally, we compared two attenuation indices for nonnecrotic lymph nodes, the nodal cortical attenuation (NCA) and the NCA-to-adjacent muscle ratio (NCA/M).

### Statistical analysis

All statistical analyses were performed using the Statistical Package for the Social Sciences Software, Version 23.0 (SPSS, IBM, Chicago, Illinois). Statistical significance was defined as P < .05. Patient age and sex, lymph node size, location (unilateral or bilateral), necrosis, perinodal infiltration, conglomeration, nodal calcification and enhancement patterns (1/2/3/4) were compared between the three groups (KD, RH, and TL) using the Kruskal-Wallis test. If a significant difference was determined among the three groups, pairwise comparisons were calculated with the Mann-Whitney U test (P < .017). The lymph node shape (L/S ratio), NCA and NCA/M were compared between the three groups (KD, RH, TL) via an ANOVA with post-hoc analysis (Tukey HSD test, P < .05). ROC analysis determined the optimal cutoff values–considering sensitivity, specificity and accuracy—for NCA/M. We also performed a multiple logistic regression analysis by the backward stepwise method, with variables eliminated at P < .1 to identify the factors discriminating the three groups (KD, RH, TL) and also two subgroups (KD and RH+TL). In multiple logistic regression analysis to discriminate KD from TL group, NCA and NCA/M values were excluded from analysis because two values were measured in only 13 patients without severe lymph node necrosis. And for discriminating KD from RH+TL group, only NCA/M value was included because NCA and NCA/M values share the same data and NCA/M value was statistically significant compared to the NCA value in separate analysis. These results were presented with adjusted odds ratios and 95% confidence intervals.

## Results

### Patient demographics

The patients’ characteristics are summarized in Table 1. There were no significant differences in the sex distribution between the three groups (KD, RH, TL), and in the age distribution between KD and RH (P = .095) as well as between RH and TL (P = .067). However, a significant difference was observed in the age distribution between KD (mean age, 27.80) and TL (mean age, 39.82; P < .017, Mann-Whitney U test).

**Table 1:** Comparison of the study populations’ characteristics and affected lymph nodes.

	KD (n = 20)	RH (n = 36)	TL (n = 34)	P value		
				KD vs. RH	KD vs. TL	RH vs. TL
Age (years), Range	18–41	14–61	17–73	0.095	0.015	0.067
Age (Mean)	27.80	33.08	39.82	-	-	-
Sex (M:F)	6:14	10:26	17:17	0.547	0.125	0.048
**Size (mm), Short**	12.65	10.72	18.71	**0.005**	**0.001**	**0.000**
**Size (mm),** Long	18.90	15.53	25.21	**0.004**	0.036	**0.000**
Shape (L/S ratio) (mean±SD)	1.50±0.27	1.45±0.22	1.37±0.23	0.735	0.141	0.356
Location (%), bilateral	6 (30.0%)	8 (22.2%)	5 (14.7%)	0.369	0.159	0.309
Location (%), Level I	6 (30.0%)	11 (30.6%)	0 (0.0%)	-	-	-
Location (%), Level II	15 (75.0%)	20 (55.6%)	16 (47.1%)	-	-	-
Location (%), Level III	16 (80.0%)	22 (61.1%)	19 (55.9%)	-	-	-
Location (%), Level IV	17 (85.0%)	19 (52.8%)	30 (88.2%)	-	-	-
Location (%), Level V	18 (90.0%)	25 (69.4%)	24 (70.6%)	-	-	-
Location (%), Level VI	1 (5.0%)	0 (0.0%)	5 (14.7%)	-	-	-
**Perinodal infiltration**	18 (90.0%)	20 (55.6%)	33 (97.1%)	**0.007**	0.306	**0.000**
Calcification	0 (0.0%)	0 (0.0%)	3 (8.8%)	-	0.241	0.109
**Conglomeration**	15 (75.0%)	13 (36.1%)	30 (88.2%)	**0.006**	0.188	**0.000**
**Necrosis**	11 (55.0%)	3 (8.3%)	32 (94.1%)	**0.000**	**0.001**	**0.000**
**Enhancement pattern (1/2/3/4)**	9/8/2/1	33/3/0/0	2/4/7/21	**0.000**	**0.000**	**0.000**

### Qualitative analysis of CT findings

A comparison of the CT findings, including size, location of the affected lymph nodes, and imaging features of the three groups is also summarized in Table 1. In the size analysis, the mean short and long diameters of the lymph nodes were significantly longer for the KD than for the RH (P = .005 (short diameter), P = .004 (long diameter)). The short diameter of the lymph nodes was significantly shorter in KD than in TL (P = .001). No significant differences were observed in the shape (L/S ratio) of the lymph nodes. The three groups’ affected nodes did not differ in the location analysis.

**Fig 6:**
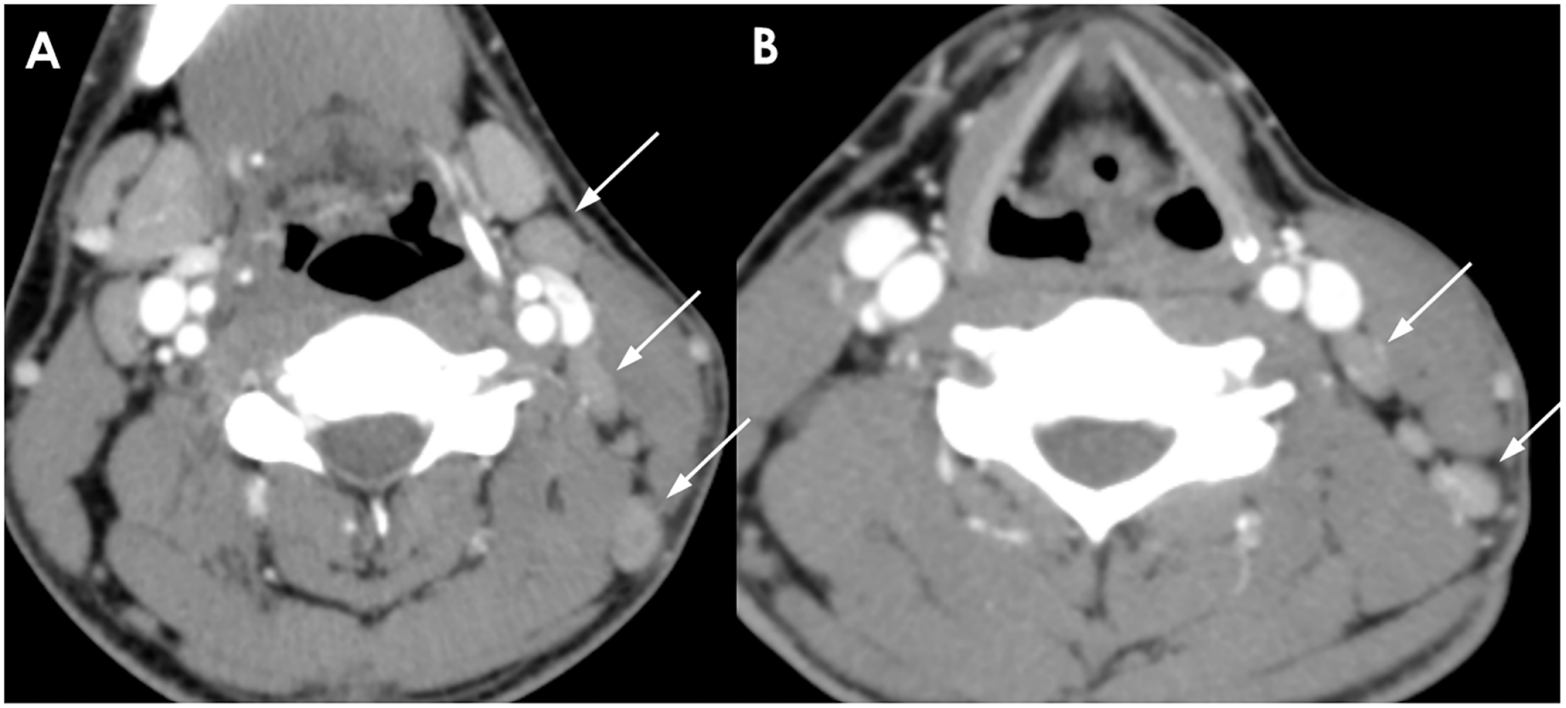
A 23-year-old man with RH. (A, B) Several enlarged lymph nodes are shown without definite conglomeration nor perinodal infiltration. Mild and homogeneous cortical enhancement is noted. The mean NCA and NCA/M are 101.0 and 1.51, respectively. The enhancement pattern for this patient is classified as group 1.

The imaging feature analysis of characteristics showed more perinodal infiltration in patients with KD than in those with RH (P = .007) and more with TL patients than in those with RH (P = .000). However, there were no significant differences between the KD and TL groups. Calcification was observed only in three patients of the TL group and conglomeration of the lymph nodes was more frequent in patients with KD than RH (P = .006) and in those with TL than RH (P = .000). No significant differences were observed between the KD and TL groups. Necrosis of the lymph nodes was observed more frequently in TL than in KD (P = .001) and RH (P = .000). In the enhancement pattern analysis, other differences were detected: Necrotic lymph nodes were more frequent in TL and nonnecrotic ones were more frequent in RH, followed by KD and TL. This suggests that in necrotic lymphadenopathy, enhancement patterns are significant CT features to distinguish TL from KD and RH. More than 50% of extensive nodal necrosis (enhancement pattern groups 3 or 4) is quite indicative of TL, which may be useful to distinguish it from KD. In more detail, extensive necrosis for KD was 15%, 45% was non-necrosis, and 40% less than 50% necrosis (Table 1, Figs 1–6).

### Quantitative analysis of CT findings

The mean NCA/M for KD, RH, and TL were 1.67 ± 0.26, 1.49 ± 0.20 and 1.47 ± 0.21, respectively. The mean NCA/M of KD was significantly higher than that of TL (P = .027) and RH (P = .011). There were no significant differences between RH and TL (P = .934). (Table 2, Figs 1–6).

**Table 2:** Comparison of the nonnecrotic portion of lymph nodes in the three groups.

	KD (n = 20)	RH (n = 36)	TL (n = 13)	P value (< .005)		
				KD vs. RH	KD vs. TL	RH vs. TL
*NCA	112.37±16.20	100.17±16.39	103.15±13.74	**0.020**	0.241	0.831
NCA/M	1.67±0.26	1.49±0.20	1.47±0.21	**0.011**^a^	**0.027**^b^	0.934

* Nodal cortical attenuation (NCA)

^a^ Threshold of NCA/M>1.60 → sensitivity 70.0%, specificity 77.8%, accuracy 72.8%

^b^ Threshold of NCA/M>1.50 → sensitivity 80.0%, specificity 69.2%, accuracy 76.2%

**Fig 7:**
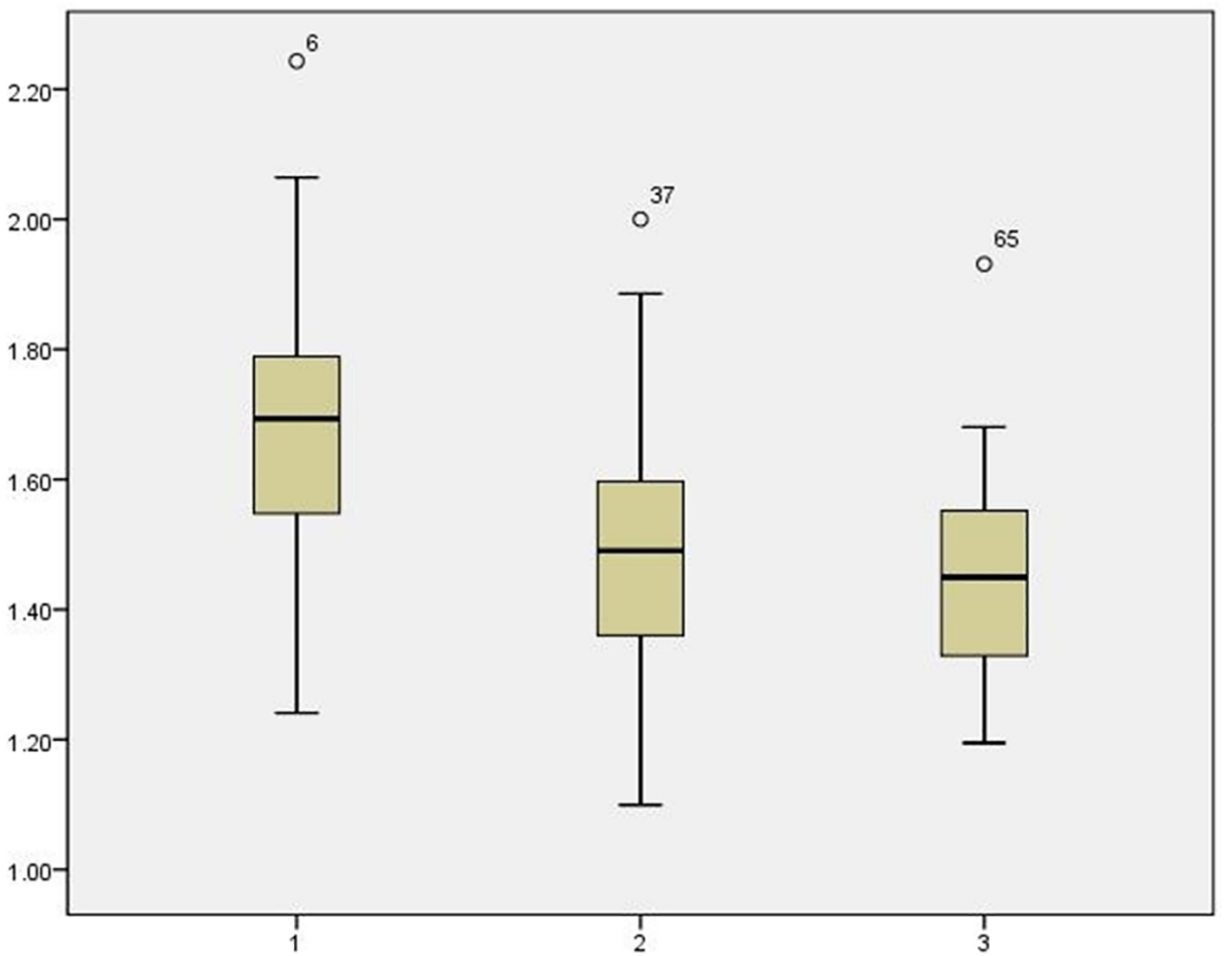
NCA/M graph of Kikuchi disease (1), reactive hyperplasia (2) and tuberculosis lymphadenopathy (3).

In the ROC curve analysis, we used cutoff values of 1.60 for NCA/M to differentiate KD from RH, yielding a sensitivity of 70.0%, specificity of 77.8% and accuracy of 72.8%. For NCA/M, the cutoff values was 1.50 to differentiate KD from TL, with a sensitivity of 80%, specificity of 69.2%, and accuracy of 76.2%. In nonnecrotic lymphadenopathy, a higher NCA/M may be more indicative for KD than RH or TL (Fig 7).

In multiple logistic regression analysis, conglomeration, enhancement pattern, and NCA/M could differentiate KD and RH, whereas the short/long diameter, perinodal fat infiltration, necrosis, NCA, and patient age could not (Table 3). Patient age and enhancement patterns differentiated KD and TL, but not the short/long diameter, perinodal infiltration, conglomeration, and necrosis (Table 4). Only NCA/M (P = .008, adjusted odds ratio 62.76) differentiated KD from RH+TL groups, but no other variables.

**Table 3:** Multiple logistic regression analysis of CT findings to discriminate KD from RH.

	P value	Adjusted Odds Ratio	95% Confidence Interval
Short diameter	0.295	1.434	0.730–2.817
Long diameter	0.648	0.923	0.656–1.301
Perinodal infiltration	0.188	4.079	0.503–33.105
Necrosis	0.999	0.000	-
NCA	0.744	0.982	0.880–1.096
Age	0.109	0.927	0.844–1.017
**Conglomeration**	**0.021**	6.717	1.327–33.997
**Enhancement pattern**	**0.012**	11.818	1.712–81.553
**NCA/M**	**0.027**	37.843	1.502–953.598

**Table 4:** Multiple logistic regression analysis of CT findings to discriminate KD from TL.

	P value	Adjusted Odds Ratio	95% Confidence Interval
Short diameter	0.233	1.269	0.858–1.877
Long diameter	0.331	0.869	0.654–1.154
Perinodal infiltration	0.787	1.708	0.035–82.380
Conglomeration	0.263	7.674	0.217–271.241
Necrosis	0.321	0.211	0.010–4.562
**Age**	**0.020**	1.104	1.016–1.199
**Enhancement pattern**	**0.000**	5.435	2.287–12.916

The NCA and NCA/M values were excluded in this logistic regression analysis.

## Discussion

TL and KD have similar clinical presentations, including palpable cervical lesions with mild tenderness, low-grade fever, malaise, and night sweat [1,4,6]. However the natural course and the therapeutic methods for each are completely different. The treatment for TL usually consists of anti-tuberculosis medication and occasional surgical removal [2,6]. KD is self-contained and the lymphadenitis resolves within one to four months [1,6]. Therefore, many previous studies have tried to find indicators for the differential diagnosis a cervical lymphadenopathy using various imaging modalities, including CT, MRI, and US [3–8]. To the best of our knowledge, no research has so far reported cortical enhancement pattern analysis targeting nonnecrotic lymph nodes separately from necrotic ones to predict KD.

Early studies reported that KD frequently affects Southeast Asian females below the age of 30 [2,4]. However, it has now been identified across all races and both genders or with minimal female predominance [5]. Our study also exhibited a slight female predominance at a 2.33: 1 ratio and a mean age of 27.8 years, which is consistent with past research. The affected lymph nodes are mostly located at level II-V and show unilateral (70.2%) and bilateral involvement (20.8%) [4], but our study displayed KD mainly in level II–V lymph nodes and common unilateral involvement (70%). Ryoo et al. observed that both mean long (L) and short (S) sizes of affected lymph nodes were significantly shorter and the shape (L/S ratio) smaller in KD than in TL [6]. However, our study showed that only the mean short diameter of the lymph nodes was significantly shorter in KD than in TL (P = .001) and no differences were observed in the shape (L/S ratio) of the lymph nodes.

In previous studies using CT, imaging findings of KD were summarized as follows: multiple, enlarged lymph nodes that are homogenously enhanced with or without necrosis and perinodal infiltration [4]. Perinodal infiltration was shown in about 90% of KD cases and this was much more frequently observed in KD than in RH (P = .007) which is by far higher rate than in previous CT studies with about 75% perinodal fat infiltration for TL [9]. Perinodal infiltration can be described to consist of four histopathologic stages in active TL and a progression beyond stage 3 indicates periadenitis. Capsular destruction of the affected lymph node is a significant feature of periadenitis, which can potentially obliterate their fat planes [10,11].

Nodal necrosis was reported in about 16.7% of KD patients [4]. Other studies found that KD patients showed no or minimal nodal necrosis and marked perinodal infiltration in contrast to the marked extent of nodal necrosis in TL [4,12]. Furthermore, Lee et al. reported that ill-defined margins of a necrotic lesion were an indicator for KD diagnosis [2]. Such results, based on pathological findings document that the extent and frequency of nodal necrosis belong to the features that distinguish KD from TL. In the histopathology, KD presents irregular paracortical coagulative necrosis with prominent karyorrhectic debris and eosinophilic fibrinoid deposits. A large number of histiocytes and immunoblasts surrounds the margin of the necrotic foci [13] which may explain the indistinct nodal architecture of KD [2,13]. However, TL typically exhibits a central low-attenuated area with rim enhancement corresponding to the central caseous necrosis surrounded by multiple granulation tissues [2,9,10].

Actually, the necrotic lymph nodes were more frequently observed in TL than in KD (P = .001) and more in KD than in RH (P = .000). There are three histopathologic stages in KD: proliferative, necrotizing and xanthomatous [13]. Various cells including eosinophilic apoptosis debris comprise the proliferative stage. Next, cellular aggregates in affected lymph nodes with any extent of coagulative necrosis may be classified as the necrotizing stage. Finally, if abundant foamy histiocytes are present, it is classified as xanthomatous with or without necrosis. In this study, the necrotizing stage was most common, observed in more than half of the cases. These histopathological types are considered to be sequentially evolving stages rather than independent subtypes [6]. However, the histopathology of TL shows early caseous necrosis with tissue destruction by pathogens [14] which may explain why necrotic lymph nodes are more frequent and extensive in TL.

Concerning the nonnecrotic lymph nodes, a higher NCA/M index could differentiate KD from TL which can be explained by the initial proliferative stage of KD before the necrotizing stage consisting of various histocytes, plasmacytoid monocytes, lymphoid cells with karyorrhectic debris and brightly eosinophilic apoptosis debris [13]. A comparison of cytological features indicates that TL has features overlapping with KD, such as necrotic materials, karyorrhectic debris and acidophilic cells [15]. However, the presence of neutrophils, epithelioid histiocytes and cheese-like necrosis is more common in TL [15]. These cytological differences may explain the higher nodal cortical attenuation in KD. In addition, a higher CT attenuation also appeared even in the necrotic portion of KD patients. This may be explained by the brightly eosinophilic fibrinoid deposits in those necrotic portions [2] and the water loss with the resulting cytoplasm concentration and protein denaturation [5].

There were some limitations to our study. First, it was a retrospective study, and there may have been a selection bias, since only patients were included who underwent contrast-enhanced neck CT and a pathological diagnosis by US-guided FNAB, core needle biopsy, or surgical excisional biopsy. Also, the sample size was relatively small. Second, since a small ROI was used to measure the nodal cortical attenuation in the affected lymph node avoiding hilar vessels, the possibility of a technical error could not be ruled out. Finally, CT scanners with three different resolutions (16, 64, and 128-detector CT) were used with the possibility of technical artifacts such as beam hardening and partial volume artifacts.

In conclusion, our results indicate that the demographic characteristics, distribution, size and shape of lymph nodes are not specific findings to differentiate KD from TL and RH. The perinodal infiltration and conglomeration of the affected lymph nodes were features contributing to a diagnosis of TL or KD rather than RH, and necrosis was more frequently observed in TL. However, the high cortical CT attenuation combined with an indistinct nodal architecture was a supportive imaging finding in lymph nodes with KD. In the case of nonnecrotic lymphadenopathy, a higher NCA/M index could potentially differentiate KD from TL.
